# Recent Advances and Clinical Application of Color Scanning Laser Ophthalmoscope

**DOI:** 10.3390/jcm10040718

**Published:** 2021-02-11

**Authors:** Hiroto Terasaki, Shozo Sonoda, Masatoshi Tomita, Taiji Sakamoto

**Affiliations:** 1 Department of Ophthalmology, Kagoshima University Graduate School of Medical and Dental Sciences, Kagoshima 890-8544, Japan; sonosho0110@gmail.com (S.S.); tommymasa0506@gmail.com (M.T.); tsakamot@m3.kufm.kagoshima-u.ac.jp (T.S.); 2 Kagoshima Sonoda Eye & Plastic Surgery Clinic, Kagoshima 890-0053, Japan

**Keywords:** color SLO, scanning laser ophthalmoscope, color fundus photography, multicolor, review

## Abstract

Scanning laser ophthalmoscopes (SLOs) have been available since the early 1990s, but they were not commonly used because their advantages were not enough to replace conventional color fundus photography. In recent years, color SLOs have improved significantly, and the colored SLO images are obtained by combining multiple SLO images taken by lasers of different wavelengths. A combination of these images of different lasers can create an image that is close to that of the real ocular fundus. One advantage of the advanced SLOs is that they can obtain images with a wider view of the ocular fundus while maintaining a high resolution even through non-dilated eyes. The current SLOs are superior to the conventional fundus photography in their ability to image abnormal alterations of the retina and choroid. Thus, the purpose of this review was to present the characteristics of the current color SLOs and to show how that can help in the diagnosis and the following of changes after treatments. To accomplish these goals, we will present our findings in patients with different types of retinochoroidal disorders.

## 1. Introduction

Examinations of the ocular fundus by ophthalmoscopy has been performed since the invention of the ophthalmoscope in 1851 and recording the color fundus photographs was introduced around 1920. Since then, color fundus photography has been used as a documenting device, and the photographs have been used for diagnosing not only ocular but also systemic disorders, e.g., arterial sclerosis, diabetes. [1,2] Color fundus photography has become indispensable for public health assessments including its use in health examinations, clinical practice, and research. However, color fundus photography has limitations such as the image quality being dependent on the pupil diameter, susceptibility to opacities of the media, and a narrow field of view of about 50°.

Scanning laser ophthalmoscopes (SLOs) with multiple laser wavelength sources, or “color SLOs”, were developed by several companies. The characteristics of these color SLOs include the use of multiple wavelength lasers as the light source, confocal technology, and advances in their image acquisition abilities. These improvements have made it possible to obtain clearer fundus images than conventional color fundus photographs. However, there have not been many clinical studies or reviews regarding the advantages and disadvantages of color SLOs. 

Thus, the purpose of this review was to present the features of the more recent color SLOs in detail. To accomplish this, we will present the SLO findings in cases of retinal disorders. 

### 1.1. History of SLOs

The SLO was first reported as a “Flying spot TV ophthalmoscope” by Webb et al. in 1980 [3]. In clinical practice, the Rodenstock SLO (SLO-101, Rodenstock, Munich, Germany) was introduced in 1990. This instrument used four wavelengths lasers and had a minimum resolution of 10 to 15 μm, which was similar to that of recent color SLOs. In addition, it was used not only as a fundus imaging device but also used as a fluorescein angiography (FA) [4,5] fundus autofluorescence (FAF) imaging [6], and blood flow measurement [7,8] and perimetry [9,10] device. However, it was not widely used because of its inferiority to the conventional fundus cameras in terms of resolution, physical size, and price.

The first widely used SLO instrument was the Heidelberg Retina Angiograph, the HRA (Heidelberg Engineering, Heidelberg, Germany), which used a high-powered laser and highly sensitive detectors with a smaller confocal aperture than conventional SLOs. This made it possible to record clear images during FA [11]. It was also possible to perform indocyanine green fundus angiography (ICGA) at the same time. Thus, HRA is still used worldwide, especially for the diagnosis of age-related macular degeneration (AMD) where the FA and ICGA findings are crucial [12].

### 1.2. Recent Advances of Color SLO

The third boom for SLO as an ocular fundus imaging device started in 2013 by the introduction of the SPECTRALIS MultiColor SLO created by Heidelberg Engineering [13]. One advantage of this device was its excellent image quality, which was proven in fundus angiography. In addition, it could be installed together with the HRA and it introduced a new method of combining color images from the images obtained by three different wavelength laser sources. Subsequently, different companies launched new SLOs, e.g., Mirante (Nidec, Gamagori, Japan) and CLARUS (Carl Zeiss Meditec Inc., Dublin, CA, USA). The ultra-widefield fundus camera (Optos PLC, Dunfermline, UK) was released in 2011 and is also an SLO that can record a pseudocolored fundus image with lasers of red and green wavelengths. [14]. In addition, a confocal light-emitting diode-based retinal imaging system (Eidon, Centervue, Padova, Italy), which is a similar concept with the color SLO, was also launched [15,16].

### 1.3. Characteristics of New SLO Devices

**Figure 1:**
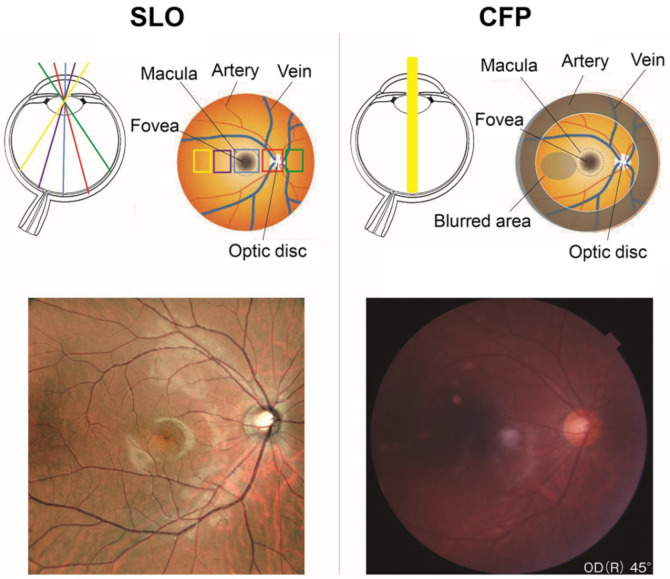
Images of a fundus of a normal eye taken by a scanning laser ophthalmoscope (SLO, **left**) and a color fundus camera through a non-dilated pupil (CFP, **right**). The subject was a 35-year-old healthy man. The fundus photograph shows a darker area on the temporal side of the macula, while the SLO records a clearer image over a wider area than the fundus camera. The SLO image was taken with the Mirante. The figure was modified with permission from Rinsho Ganka 75(1):11–19, 2021.

There are several characteristics of the new SLO devices that make them more helpful for ophthalmologists. First, color SLOs can obtain clearer ocular fundus images than conventional fundus cameras even from non-dilated eyes. The conventional fundus cameras record a fundus image by using a flash-lamp source containing different wavelengths, and the film or detector receives the light reflected from the ocular structures. The images taken by conventional fundus cameras are degraded by media opacities, pupillary diameter, and cataracts. In normal, non-dilated eyes, the amount of light entering the eye is reduced, which makes it more difficult to obtain bright and clear images (Figure 1). 

**Figure 2:**
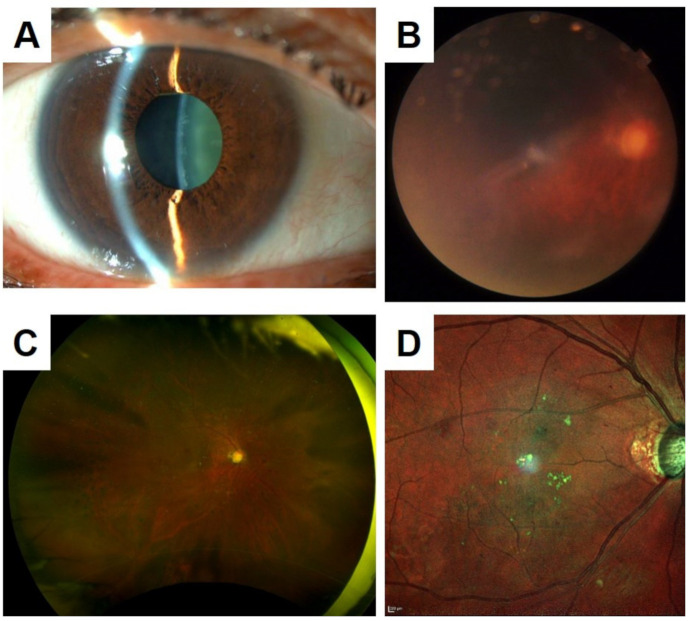
Comparison of color fundus photographs recorded with a conventional fundus camera to that obtained with a SLO device in patients with a cataract and poor mydriasis. (**A**) Fundus photograph of a 65-year-old man with poor mydriasis and cataracts. The color fundus photograph is blurred (**B**), but the ultra-widefield fundus images (Optos California, **C**) and color SLO (MultiColor, **D**) provide clearer images.

SLO uses laser light sources that scan a small area of the ocular fundus at high speed, and the reflected light signal is detected by a recording device. In principle, the laser beam irradiates the fundus of the eye through the same area of the pupil plane so a clear image can be obtained without mydriasis (Figure 1). In addition, lasers with longer wavelengths are less susceptible to corneal opacities and cataracts, and they can record clearer fundus images than conventional fundus cameras in eyes with media opacities (Figure 2).

**Figure 3:**
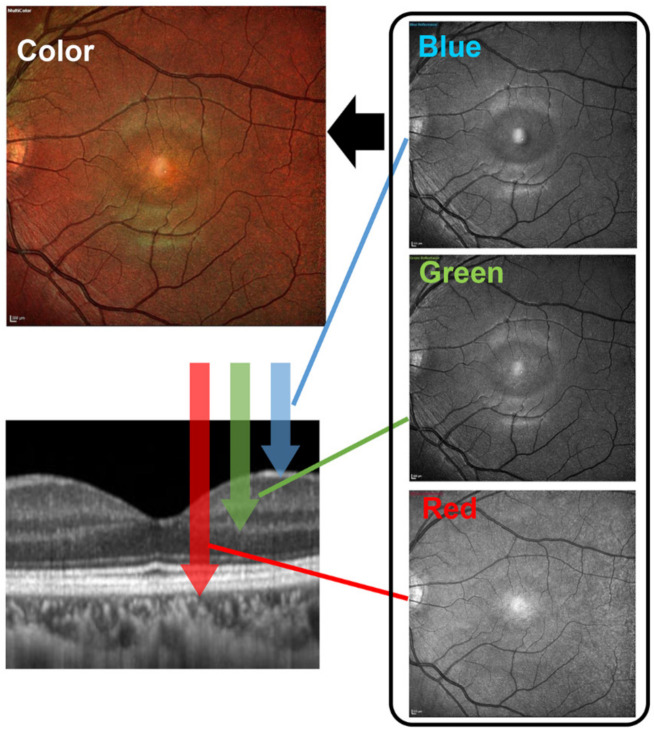
Schematic diagram of the mechanism of color scanning laser ophthalmoscope. The color SLOs use two or three different wavelength light sources to obtain an image of the ocular fundus. Each wavelength reaches different layers of the retina. The short wavelengths (blue) produce images of the superficial layer of the retina, intermediate wavelengths (green) produce images of the intermediate layers, and long wavelengths (red) produce images of the deeper layers of the retina. Thus, each wavelength produces a distinctive image of the different layers of the retina. A full color SLO image can be obtained by merging the individual wavelength images.

Second, color SLOs can obtain better quality fundus images than conventional fundus cameras. Unlike the conventional fundus camera that uses a light source containing different wavelengths, the recently introduced color SLOs use lasers of two or three different wavelengths to obtain an image of the ocular fundus. Each wavelength reaches a different layer of the retina (Figure 3). The short wavelengths (blue) produce images of the superficial layer of the retina, the intermediate wavelengths (green) produce images of the intermediate layers, and long wavelengths (red) produce images of the deeper layer of the retina. Importantly, each wavelength produces a distinctive image of the different layers of the retina [13]. The width of a scan is about 10 μm but varies with the model [17]. This is about the same as the 6 × 6 mm scan of recent Optical coherence tomography (OCT) devices, making it possible to record changes in a small area. The color SLO superimposed images of those taken by each wavelength are not only of better image quality but also provide more information at different depths in the retina than conventional fundus cameras.

**Figure 4:**
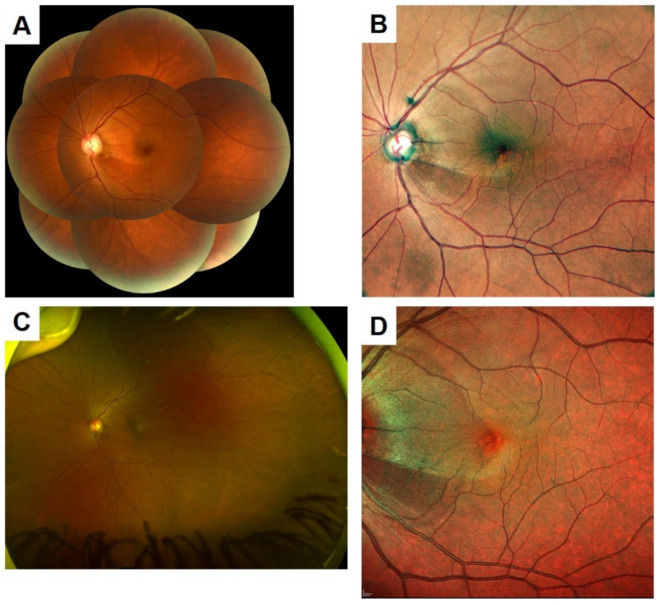
Comparisons of color fundus montage image and ultra-widefield SLO images. The images are of a 57-year-old woman undergoing treatment for normal tension glaucoma. A montage of color fundus photographs (**A**) provides a wider field image than the regular color SLO images (**B**: Mirante, **D**: MultiColor), but the image preparation was time consuming and required manpower. The ultra-widefield SLO images (**C**: Optos California) are even wider and can be recorded in a single imaging session. Note that the nerve fiber layer defect is more visible in the color SLO images (**B**,**D**) than in the color fundus photograph (**A**).

Finally, color SLO can obtain images of a larger area of ocular fundus than conventional fundus cameras. If a larger area of the fundus is needed with color fundus camera, a montage of images obtained from multiple shots can be done. However, this requires the patient to move their eyes, which can be difficult in the elderly patients, montage requires specialized software, and the creation of images takes a lot of manpower and time (Figure 4A).

**Figure 5:**
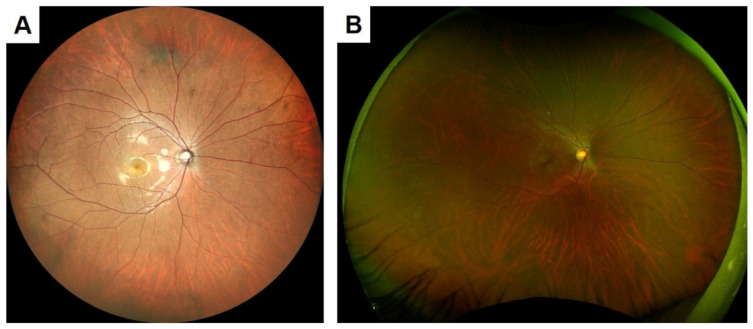
Ultra-widefield images obtained by color SLO devices. The images from the same case as in Figure 1 were taken without mydriasis. The imaging device was Mirante in (**A**), which was used with permission from Rinsho Ganka 75(1):11–19, 2021, and Optos California in (**B**).

Recent SLO devices have replaceable lenses that can be used to obtain wider angle images and an automatic image montage function that makes wide-angle imaging easier (Figure 5A). In addition, the SLO devices from Optos can produce ultra-widefield fundus images by taking advantage of the features of SLO of two wavelengths (Figure 5B). The widefield fundus images of a healthy eye taken with the Mirante and Optos California are shown in Figure 5.

## 2. Recent Clinical Studies Using Color SLOs and the Effects on Clinical Practice

In recent years, a number of studies have demonstrated the usefulness of color SLO in diagnosing and treating retinal diseases. In the AMD-related findings, geographic atrophy [18], polypoidal choroidal vasculopathy [19], subretinal fluid, and photoreceptor layer loss were reported to be recorded more clearly in the color SLO images than conventional color fundus photographs [13,19]. In central serous chorioretinopathy, color SLO was not only able to demonstrate the subretinal fluid more clearly than in the color fundus photographs recorded with the conventional cameras, but also to confirm the leakage points in about 90% of the cases compared to about 30% in the conventional fundus photographs. These findings indicate the usefulness of color SLO [20]. Govindahari et al. compared the findings of color SLO and autofluorescence in detecting macular telangiectasia. They found that the fundus findings related to Mactel, such as retinal crystals, retinal atrophy, intraretinal pigment hyperplasia, and retinal pigment epithelium (RPE) atrophy, which only appeared as hyperfluorescence or hypofluorescence in the FAF images, were recorded with specific and discriminative findings in the color SLO images [21].

As described, color SLO is helpful in detecting abnormal findings in eyes with retinal diseases. Furthermore, some reports have investigated how it is applied in clinical ophthalmology. For example, the authors found that epiretinal membrane (ERM) findings were not only clearly displayed in the color SLO images, but also that medical residents were able to determine ERM findings with high detection rates similar to that of retinal specialists [17]. In addition, Zhang et al. reported that they were able to shorten the time for ERM peeling and improve the outcomes by simulating the surgery using color SLO before the actual surgery [22].

On the other hand, there have been several reports investigating the artifacts seen at the posterior pole in the color SLO images. [23,24,25] The type and frequency of artifacts varied depending on the SLO instrument, but this issue should be considered in the analyses of color SLO images.

## 3. Representative Cases

We will present ocular fundus photographs of representative retinal disorders that clearly depict the alterations of the retina as seen in the color SLO images.

### 3.1. Retinal Nerve Fiber Layer (RNFL) Defects

Defects in the retinal nerve fiber layer are the pathological findings in glaucomatous optic neuropathy, and these defects can precede the changes in the optic disc and visual field [26]. Thus, the detection of RNFL defects can be a diagnostic tool for early glaucoma [27,28]. 

**Figure 6:**
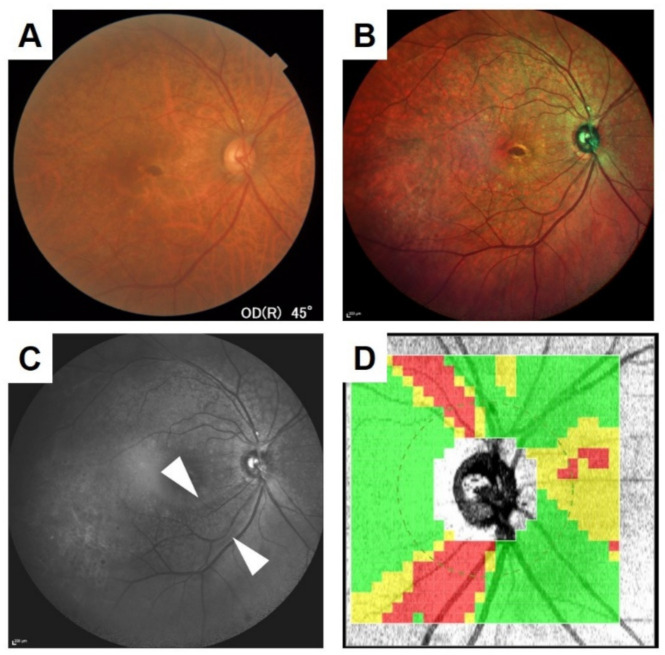
Findings of a 65-year-old woman with open-angle glaucoma and age-related macular degeneration. The retinal nerve fiber layer (RNFL) defect is difficult to detect in the ocular fundus image obtained by a conventional fundus camera (**A**), but the defect is easily detected in the blue wavelength image obtained by color SLO (**C,** arrow head). Composite image of color SLO (**B**) and OCT thickness map (**D**). The color SLO image was obtained by a MultiColor. The figure was modified with permission from Rinsho Ganka 75(1):11–19, 2021.

The ocular fundus of Asian eyes has an orange-colored appearance and RNFL defects have a brown tone. Thus, defects in the RNFL tend to be missed in Asians due to the low contrast between them and the surrounding retina. The images obtained by the SLO with blue wavelength laser light is helpful and easier for detecting the RNFL defects [29]. The RNFL defects are relatively difficult to detect in the images obtained by conventional fundus photography, as seen in a representative case in Figure 4 and Figure 6. The authors reported that they detected the RNFL defects from the color SLO images in patients with retinal diseases who had no glaucomatous changes in the optic nerve in the images obtained by the conventional fundus photography [29]. Such conclusions were confirmed in cases of AMD, macular hole after vitrectomy, and microaneurysms (MAs) after photocoagulation.

### 3.2. Epiretinal Membrane (ERM)

**Figure 7:**
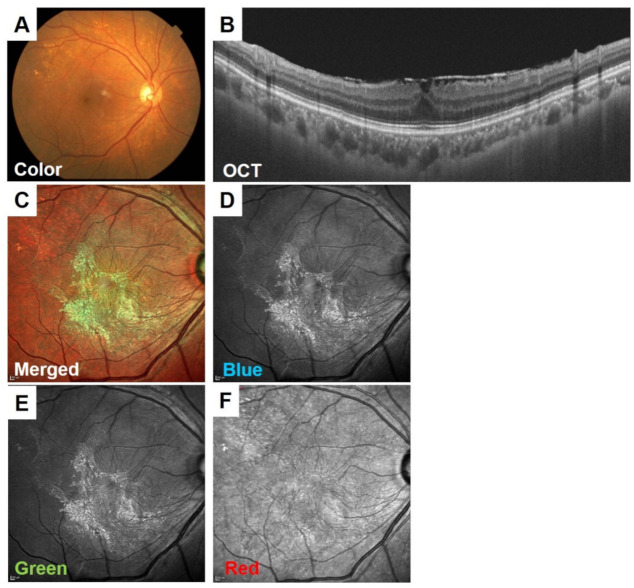
Fundus images of a 70-year-old woman with an ERM. Although the ERM is not clearly visible in the conventional color fundus photograph (**A**), it can be seen in the OCT images (**B**). In the color SLO images (**C**–**F**, MultiColor), the ERM membrane is visible in the blue wavelength (**D**) and green wavelength (**E**) images. The retinal folds are also visible in the merged color images (**C**) and red wavelength image (**F**).

An ERM is another relatively common disorder that can be clearly seen in color SLO images even though the ERM is not visible in the conventional fundus photographs [17,18]. Although color SLO images tend to focus on color images composed of images of a single wavelength, our study found that the fibrotic membranes of ERMs were more visible in short wavelength images in green and blue, which provided greater contrast with the background (Figure 7) [17]. On the other hand, the retinal fold component was more prominent in the red wavelength SLO images and also in the combined color images. The retinal folds, which are superficial retinal findings, are believed to “show up” in the red wavelength images, which images the structures in the deeper retinal layers. Color SLO images are useful for non-retina specialists to see the findings of ERMs and for an explanation of the disorder to patients.

## 4. Retinal Capillary Microaneurysms

**Figure 8:**
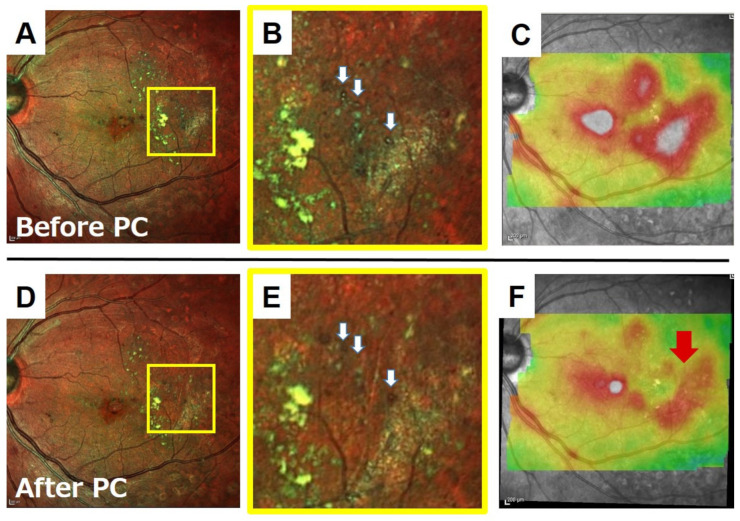
A case in which direct photocoagulation of microaneurysms (MAs) was performed with guidance from color SLO images. The patient was a 56-year-old woman with diabetic macular edema due to MAs. Prior to treatment, color SLO and retinal thickness map obtained by OCT B-scan were used to identify the MAs on the lateral side of the macula (**A**–**C**), and photocoagulation was performed (**B**, white arrows). One month after the treatment, the edema at the coagulation site was improved and hard exudates were reduced (**D**–**F**, red arrow). **B** and E are magnified images of the yellow square in **A** and **D,** respectively. Color SLO was taken with a MultiColor.

Retinal capillary microaneurysms (MAs) are usually the first visible sign of diabetic retinopathy. MAs are identified by ophthalmoscopy as red dots varying from 25 μm to 100 μm in diameter. They are commonly found in the posterior pole of the eye [30]. MAs have been recorded in either a green-red, a red, or a green pattern in composite images of color SLO images [31]. Although the first choice of treatment for diabetic macular edema (DME) is anti-vascular endothelial growth factor (VEGF) drugs, laser photocoagulation of MA is useful in resolving the retinal edema that is caused by MAs [32]. Although the gold standard for detecting MAs is FA, patients with MAs are more likely to have systemic diseases, such as diabetes and hypertension, and it is difficult to perform FA frequently on them. The detection of MAs by color SLO is easier than by conventional fundus cameras, and it is helpful in using color SLO images for direct coagulation of MAs (Figure 8).

### 4.1. Retinal Vein Occlusion (RVO)

**Figure 9:**
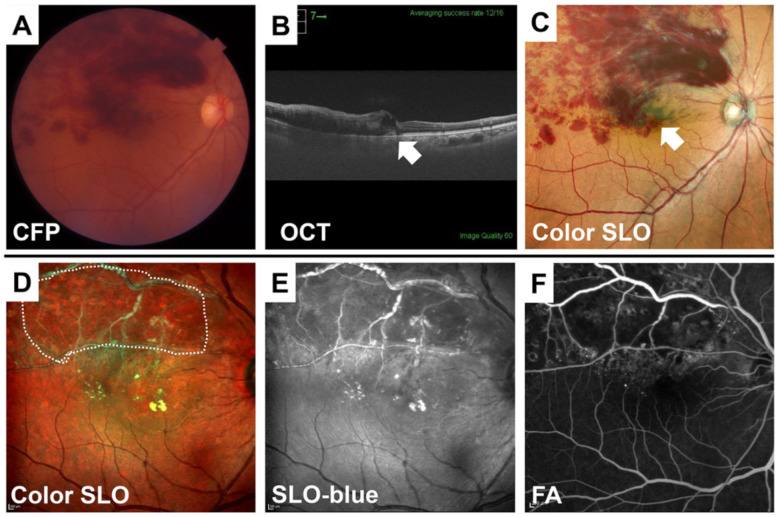
Color SLO images of eyes with BRVO at the acute and chronic phase. BRVO is easy to diagnose by conventional fundus photographs because of the prominence of retinal hemorrhages (**A**). Color SLO images can further distinguish between the presence of macular edema and retinal and subretinal hemorrhages (**B**,**C**, white arrow). In chronic stage of BRVO, it is easy to distinguish the whitening of the blood vessels and non-perfused areas that are darker in the SLO images, especially with blue wavelength images (**D**,**E**, white dot circle). This is consistent with the nonperfused areas in fluorescein angiographic image (**F**). Color SLO was taken with a MultiColor.

A RVO is caused by the blockage of a branch of a retinal vein, which leads to retinal hemorrhages, occasionally subretinal hemorrhages, and macular edema. Branch retinal vein occlusion (BRVO) is easy to diagnose by conventional fundus photographs because of the presence of retinal hemorrhages. Color SLO images can further distinguish between the presence of macular edema and retinal and subretinal hemorrhages (Figure 9). In chronic-stage BRVO, it is easier to distinguish the whitening of the blood vessels and non-perfused areas that are darker in the SLO images recorded with blue wavelengths than that in the conventional fundus photographs.

### 4.2. Central Serous Chorioretinopathy (CSC)

**Figure 10:**
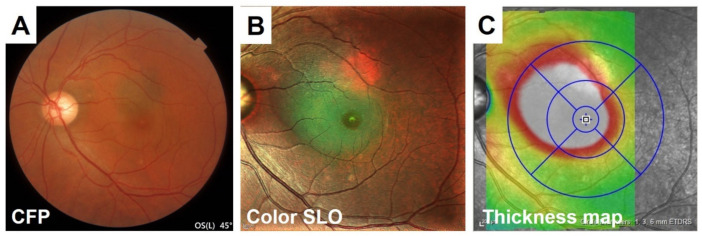
Comparison of the detection of serous retinal detachment in a CSC patient between conventional color fundus photographs (CFP) and color SLO images. Although the conventional fundus photograph can detect the serous retinal detachment (SRD) at the fovea, it can sometimes be missed because the color change at the SRD site is not so sharp (**A**). It is relatively easy to diagnose CSC in the color SLO images because of the color change in the area associated with SRD, which is comparable with OCT thickness map and is larger than that of color fundus photographs (**B**,**C**). Color SLO was taken with MultiColor.

CSC is a retinochoroidal disorder that is associated with a serous retinal detachment (SRD) in the macula. CSC is now widely considered to be one of the pachychoroidal disorders [30]. The detection of an SRD is important to correctly diagnose CSC because the absence of SRD is now considered a pachychoroid pigment epitheliopathy [33]. Although conventional fundus photographs can be used to detect the SRD at the fovea, CSC can occasionally be missed because the color difference at the site of the SRD is not so sharp. CSC is relatively easy to diagnose in the color SLO images because the color changes in the area associated with the SRD is larger than that seen in the conventional fundus photographs (Figure 10).

### 4.3. Age-Related Macular Degeneration and Other Acquired Macular Conditions

**Figure 11:**
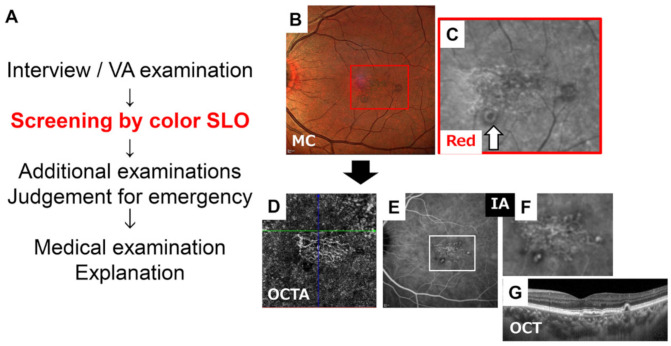
Flowchart of outpatient care utilizing color SLO. Recording the color SLO without mydriasis in advance following the interview and minimal examination such as the visual acuity (VA) check will improve the efficiency of the examination (**A**). In this case of AMD, it is possible to diagnose this case as an AMD by color SLO image, especially in the red wavelength images that show the vascular network and suspected polyps (**B**,**C**). Then, the clinician can make plans of the next needed examinations. In this case, OCT angiography (**D**), indocyanine green angiography (**E**,**F**) and OCT B-scan around macular area with small interval (**G**) to validate the diagnosis were performed. (**C**) is a magnified red wavelength image of red square in (**B**). (**F**) is a magnified image of white square in (**E**). Color SLO was imaged with the MultiColor. The figure was modified with permission from Rinsho Ganka 75(1):11–19, 2021.

Color SLO imaging is also helpful in identifying AMD because the pathological changes are present in the retinal pigment epithelium and the choroid. Thus, images obtained with red wavelengths, which are simultaneously taken with OCT scan, are helpful in detecting the abnormal findings in patients with AMD and other acquired macular conditions. [34,35] The choroidal vascular network and pigment epithelial detachments can be seen in the images taken by red wavelength and merged color SLO images, while these findings are undetectable in the conventional fundus photographs (Figure 11).

## 5. Conclusions

We concluded that color SLO images will be of great help for clinicians because it allows various ocular fundus findings to be seen in a single en face image. The quality of the image is better and is larger. The color SLO images incorporate information at different retinal depths and, once the examinee becomes accustomed to them, it is possible to detect less obvious pathological findings, which are difficult to detect in conventional fundus photographs. In the future, it may be possible to reduce the risk of missing abnormal findings and improve the efficiency of medical care by taking color SLO images without mydriasis for diagnosis before a detailed examination and then including the appropriate tests (Figure 11).

Most of the studies using color SLO have reported higher rates of detection of retinal abnormalities compared to conventional color fundus photographs. In addition, there are also reports of clinical contributions by enabling the making of a correct diagnosis by non-specialists [17] and improving surgical performances [22]. Recently, there has been a lot of research on artificial intelligence (AI) using fundus photographs, OCT, and OCT angiography images [36,37,38,39,40], and there is a highly likelihood that color SLO, which has high image quality with a large amount of information, will be useful for this kind of automatic diagnosis by AI. Indeed, preliminary studies using AI and color SLO have been reported [16,41,42].

It will be necessary to assess whether the results will be superior to those of AI diagnosis using conventional fundus photographs and whether they will contribute to the clarification and finding of new pathological changes in retinochoroidal diseases.

## Data Availability

No new data were created or analyzed in this study. Data sharing is not applicable to this article.
